# Investment in Constitutive Immune Function by North American Elk Experimentally Maintained at Two Different Population Densities

**DOI:** 10.1371/journal.pone.0125586

**Published:** 2015-05-20

**Authors:** Cynthia J. Downs, Kelley M. Stewart, Brian L. Dick

**Affiliations:** 1 Department of Natural Resources and Environmental Sciences, University of Nevada, Reno, Nevada, United States of America; 2 Pacific Northwest Research Station, United States Forest Service, La Grande, Oregon, United States of America; Sonoma State University, UNITED STATES

## Abstract

Natural selection favors individuals that respond with effective and appropriate immune responses to macro or microparasites. Animals living in populations close to ecological carrying capacity experience increased intraspecific competition, and as a result are often in poor nutritional condition. Nutritional condition, in turn, affects the amount of endogenous resources that are available for investment in immune function. Our objective was to understand the relationship between immune function and density dependence mediated by trade-offs between immune function, nutritional condition, and reproduction. To determine how immune function relates to density-dependent processes, we quantified bacteria killing ability, hemolytic-complement activity, and nutritional condition of North American elk (*Cervus elaphus*) from populations maintained at experimentally high- and low-population densities. When compared with elk from the low-density population, those from the high-density population had higher bacteria killing ability and hemolytic-complement activity despite their lower nutritional condition. Similarly, when compared with adults, yearlings had higher bacteria killing ability, higher hemolytic-complement activity, and lower nutritional condition. Pregnancy status and lactational status did not change either measure of constitutive immunity. Density-dependent processes affected both nutritional condition and investment in constitutive immune function. Although the mechanism for how density affects immunity is ambiguous, we hypothesize two possibilities: (i) individuals in higher population densities and in poorer nutritional condition invested more into constitutive immune defenses, or (ii) had higher parasite loads causing higher induced immune responses. Those explanations are not mutually exclusive, and might be synergistic, but overall our results provide stronger support for the hypothesis that animals in poorer nutritional condition invest more in constitutive immune defenses then animals in better nutritional condition. This intriguing hypothesis should be investigated further within the larger framework of the cost and benefit structure of immune responses.

## Introduction

Natural selection favors individuals that respond with effective and appropriate immune responses to macro or microparasites [1, 2]. Vigorous immune function might be best for fending off parasites, but also may lead to immunopathology or diversion of resources from growth or reproduction [3–5]. Conversely, if immune function is not sufficient to clear or resist the parasite and if an individual does not tolerate a parasite, animals might succumb to disease or parasites [3, 4, 6]. Indeed, optimal immune responses that balance costs and benefits are strongly correlated with survival (e.g., [7, 8]).

Included within the equation that determines optimal immune defense are the energetic and nutritional costs of maintaining and mounting immune responses [9–11]. Those costs add demands for energy and nutrients above the cost of maintaining the body, which may result in diversion of resources from production of tissues during growth or reproduction [11–13]. Tissue production is most likely to be compromised if food supply is low or food intake is reduced by endogenous processes (such as anorexia associated with an acute phase response [14]) that suppress appetite or impair digestion and absorption [15, 16]. Individuals in populations at high density relative to ecological carrying capacity experience reduced food availability because of increased intraspecific competition [17–20]. As a result, individuals in high-density populations exhibit poorer nutritional condition, lower pregnancy rates, lower recruitment rates, older age at first reproduction, and are more likely to pause in annual reproduction than those in low-density populations [17, 20–23]. Within this framework, individuals in high-density populations also may invest sub-optionally in immune function.

As more individuals occur on a landscape they occur in larger groups and are closer together, and this close proximity of individuals to one another also favors transmission of disease ([24, 25, 26] but see exceptions therein). This effect also could lead to density-dependent prophylaxis—preemptive increases in investment in constitutive immunity triggered by increased population density—as observed in insects [27, 28]. Alternatively, investment in immune function might increase in high-density populations because of increased transmission of parasites leading to increased parasite loads [1]. Costs of immunity and repair are thus part of density-dependent feedbacks, which also are manifested as reduced growth of the population by slowing rates of reproduction and growth of juveniles, as well as increasing rates of mortality, which ultimately slows population growth [17, 20, 21, 29]. Investigations of the relationship between population density and physiological responses have primarily focused on small rodents in captive studies or natural irruptions [15, 24, 30–33]. Herein, we present a study system that uses long-term, experimental manipulation of a population of large mammals at large spatial scales to investigate how population density affects investment in constitutive immune responses.

Constitutive immune responses are always present and are capable of immediate physiological defense [34], and investment in constitutive immune responses may be quantified in several ways including two effective and related assays: bacteria killing ability and hemolytic-complement activity [35]. The bacteria killing assay assesses the ability to eliminate an ecologically relevant pathogen, and thereby provides a functionally relevant assessment of host immune function. Specifically, this assay characterizes an immune response that involves the action of opsonizing proteins (mainly complement, but also acute phase proteins) and natural antibodies (predominantly IgM and IgA [35, 36]). Natural antibodies are the constitutive repertoire of antibodies that are present in small concentrations and circulate regardless of previous exposure to a parasite [2]. Because of the bacteria we used in the bacteria killing assay, we are mainly quantifying complement levels. The hemolytic-complement activity assay quantifies overall pathway integrity, cell lysis, and functional activity of the complement pathway [36, 37], a part of the innate immune response that consists of a series of proteins present in the serum [38].

Our objective was to understand the relationship between immune function and density dependence as mediated by correlations among immune function, nutritional condition, and reproduction using North American elk (*Cervus elaphus* Linnaeus). As a long-lived iteroparous species, elk have evolved strategies for energy allocation that maximize reproductive success over their lifetime by placing less emphasis on any single reproductive event [39–42]. To test how immune function relates to reproductive status (pregnancy and recruitment) and population density, we used North American elk from populations maintained at experimentally high and low population densities on the Starkey Experimental Forest and Range. Elk in the high-density population at Starkey exhibit classic density-dependent responses in reproductive traits, and those responses are driven by changes in nutritional status caused by increased intraspecific competition on summer range [17]. We hypothesized that population density affects investment in immune function and we predicted that individuals from high-density population have lower immune responses, because they have fewer resources available to invest in costly immune function. Within this framework, we also hypothesized that individuals will not allocate resources away from immune function in early stages of pregnancy. Thus, we predicted that pregnant individuals have higher immune function than those that are not pregnant. When resources are limiting, females that successfully recruit an offspring are typically in poorer nutritional condition entering winter than those that did not successfully recruit an offspring [17]; thus, we predicted that females that successfully recruited an offspring have lower nutritional status and lower immune function than non-reproductive females entering winter. Finally, yearling elk are still investing resources into growth and rarely are reproductive [43]; therefore we predicted that yearlings have lower immune functions than adult elk.

## Materials and methods

### Study Area and Experimental Design

We conducted research during winter 2012–2013 on the Starkey Experimental Forest and Range (hereafter, Starkey 45°12′N, 118°3′W). Starkey is managed as a formal research site by the USDA Forest Service Pacific Northwest Research Station [44]. Starkey is situated in the Blue Mountains of northeastern Oregon, USA, and is located about 35 km southwest of La Grande, Oregon, USA [17, 45]. Starkey is an ideal location to test questions about how investment in immune function correlates with life histories because extensive data are collected, including nutritional condition and reproductive status, and maintained on the majority of individuals in the study areas.

Starkey encompasses 10,125 ha, and elevations range from 1,120 to 1,500 m. Since 1987, Starkey has been surrounded by a 2.4-m fence that prevents immigration or emigration of large herbivores, including migration to traditional winter ranges [44, 46]. During the summer, elk are free-ranging in one of three study areas: main study (10,125 ha), northeast-east (842 ha), and northeast-west (610 ha). Our study was focused in the northeast (east and west) study areas, which have equal proportions of habitats to accommodate experimental comparisons of population densities of elk [17, 45]. The northeast-east study area had a high population density (11.9 elk km^-2^, 100 individuals) since 2002, while the northeast-west section had a low population density relative to carrying capacity (4.1 elk km^-2^, 24 individuals) [17, 45]. For this study, we define ecological carrying capacity as the number of animals at or near equilibrium with their food supply [21, 47].

Because elk cannot migrate to traditional winter range, they are maintained on a winter feedground. During the early winter, elk are captured in holding pastures, processed at a handling facility, and then released onto winter feedground where they are fed a maintenance diet of alfalfa hay [17]. Elk from both the high- and low-density areas were housed together on the winter feedground and experienced the same conditions over winter, thus changes in nutritional status resulted from resource availability and intraspecific competition on summer range [17]. As elk entered the feedground during winter, they were captured and moved via a system of alleys through the handling facility for collection of data on individual animals [17, 44]. Yearlings, young (<1 year old), and adult females (≥2 years old) were moved through the handling facility and each individual was identified by unique ear tags [17, 45].

We quantified nutritional status of individuals by measuring maximal depth of subcutaneous fat on the rump [17, 48–51], and determined pregnancy and lactation status. Maximum depth of subcutaneous fat on the point of the hip was measured via ultrasonography as a measure of nutritional condition [17, 48, 49]. We used palpation to determine if individuals were lactating, and we used lactational status as an index of recruitment of young that was specific to the individual female [17, 42]. Blood was collected from the jugular veins of adult and yearling females, centrifuged to separate serum from red blood cells, and serum was frozen immediately at -30°C for later analyses. One portion of the serum was analyzed for pregnancy-specific protein B (Bio Tracking, Moscow, Idaho, USA) to determine pregnancy status of individuals [17, 52]. A second portion was frozen, transported to the lab, and used for quantifying immunocompetance.

### Immunocompetance assays

We quantified constitutive immune responses using two closely related assays: a bacteria killing assay and a hemolytic-complement activity assay. Prior to performing these assays, serum was thawed and filtered with syringe filters (Acrodisc syringe filters with supor membrane, pore size of 0.2 μm, Pall Life Science) to remove hemoglobin released by lysing of red blood cells during processing of whole blood [53].

We used bacteria killing assays to measure a functional response by the constitutive, innate immune system against a relevant pathogen, *Escherichia coli* [54]. We performed assays on serum samples, following Zysling et al. [55], using *E*. *coli* (E^power^ Microorganism*s* 0483E7, ATCC 8739, MicroBioLogics, St. Cloud, MN), and a 1:40 dilution of serum samples in Luria-Bertani (LB) broth. Briefly, 5 μl of a sample was mixed with 295 μl of LB broth, and then 20 μl of *E*. *coli* solution (~5000 bacteria ml^-1^) was added. We also made two positive controls of 200 μl LB broth and 20 μl of *E*. *coli* solution. Samples and controls were vortexed and incubated at 37°C for 30 minutes. We then vortexed samples and controls again and plated 50 μl aliquots onto LB agar in petri dishes. We made three replicate plates for each sample and tested samples in batches of ≤11 samples to minimize differences in incubation time of the first and last sample plated. One of the positive controls was plated at the beginning and another one was plated at the end of each batch; each positive control was plated in triplicate. Plates were incubated overnight at 37°C, after which bacteria colonies were counted. We found no difference in the number of colonies that grew on the controls plated at the beginning and end of each batch (*F*_1,87_ = 1.29, *P* = 0.26); consequently, we used all 6 positive control plates to determine the mean number of control colonies for a particular batch. Samples were compared with the positive controls from their batch. Bactericidal capacity was calculated as one minus the mean number of colonies from each sample, divided by the mean number of colonies from the positive controls, and multiplied by 100 (i.e., % bacteria killed relative to the positive control).

Hemolytic-complement activity in serum was measured with a method adapted from Sinclair and Lochmiller [56]. Briefly, we diluted serum to a 1:40 dilution with dextrose gelatin veronal buffer (DGV, catalog # 10-539B, Lonza Inc., Allendale, NJ, USA). In a 96-well plate, we mixed 80 μl of each sample with 25 μl of 0.06% suspension of sheep red blood cells (SRBC) (Innovative Research, Novi, MI, USA) in DGV and 25 μl of a 1:40 dilution of rabbit anti-sheep red blood cell antibodies (Sigma-Aldrich, St. Louis, MO, USA, product # S1389) in DGV. Samples were analyzed in duplicate. We also used a 100% lysis control of 65 μl of deionized water and a 0% lysis control of 65 μl DGV in duplicate. We added 25 μl of diluted SRBC, but no antibodies, to both controls. The plate was shaken gently for 5 min. on a plate shaker, and then incubated for 90 min. at 37°C. The plate was then centrifuged for 5 min. at 84 rcf, and 60 ul of supernatant was transferred to a new 96-well plate. Absorbance was measured at 405 nm.

The original protocol called for determining the dilution of serum required to lyse 50% of the SRBC in culture, or CH50 [37, 56]. Ideally, one should use two dilutions of the sample that encompasses the CH50 to help ensure the points used fall on the linear part of a sigmoidal curve that represents the lysing activity in this assay. When we calibrated the assay to determine the two appropriate dilutions for calculating the CH50, we observed that variation in our serum samples precluded the use of a single pair of serum dilutions for all samples and that some samples had very low lysing ability across the range of dilutions tested. Hence, we measured hemolytic-complement activity as the percentage of SRBC lysed at a single dilution as a representation of hemolytic-complement activity. This choice limits the scope of our inferences to this single concentration but it still provided information about the hemolytic-complement activity at a single concentration and allows for inferences within a framework comparing hemolytic-complement activity of individuals. Whenever possible, we recommend that researchers use two dilutions to improve the breadth of inference that can be draw about hemolytic complement activity and to facilitate comparisons among different studies.

Prior to running our immunocompetence assays we tested a separate set of serum samples from elk to determine if freezing samples affected results of immunocompetence assays as reported in previous work quantifying bacteria killing ability in house sparrows (*Passer domesticus* Linnaeus) [57]. Details are provided in S1 Text. Briefly, our results indicated that freezing did not degrade elk serum samples; rather, the samples in the refrigerator degraded significantly during the three days required to transport them from the field site to the laboratory.

### Data Processing and Statistics:

We used generalized linear models (GLM) with an identity link function, to investigate what factors affected percent bacteria killed and percent SRBC lysed. Errors had normal distributions and estimates were fit using maximum likelihood. Population density (high or low), age class (yearling or adult), lactation status (lactating or not lactating), pregnancy status (pregnant or not pregnant), and nutritional condition (maximal depth of subcutaneous fat, a continuous covariate) were included as fixed effects for models of both response variables.

Nutritional condition might be an important mediator of investment in immune responses, but might not be significant in the models of immune function, because nutritional condition also was associated with other potential predictors of immune function including lactation status, pregnancy status, and population density [17]. Thus, we determine how nutritional condition was affected by population density, age, lactation status, and pregnancy status with a GLM fit with an identity link function. Errors were normally distributed and estimates were fit using maximum likelihood. That analysis allowed us to determine if the pattern of estimated means for nutritional condition was similar to the pattern of estimated means for immune responses. For example, if individuals from the high-density population have the lowest nutritional condition and the lowest bacteria killing ability, then these results would support the hypothesis that density causes individuals to reduce investment in immune function because density reduced nutritional condition.

For all analyses we used Procedure GENMOD in SAS [58]. We used the procedures recommended by Zuur et al. [59] to explore our data and results to ensure that were not violating assumptions of our statistical models. Specifically, during initial exploration of our models for all response variables we included interactions terms between class variables and body condition, but those interactions were not significant so they were not included in the final model. We ensured that were not violating assumptions of the models by using q-q-plots to inspect distribution of residuals, visually inspecting conditional boxplots to test for equal variances, and using Cook’s distance to check for data points with leverage [59]. Data used in our analyses are in S1 Table.

### Ethics Statement

All procedures for handling animals were in accordance with guidelines established by the American Society of Mammalogists for capture and handling of wild mammals for research [60], complied with US laws, and were approved by the Institutional Animal Care and Use Committees of the Starkey Project (Protocol #92-F-0004) and the University of Nevada Reno (Protocol #00565), which is accredited by the Association for Assessment and Accreditation of Laboratory Animal Care.

## Results

**Fig 1:**
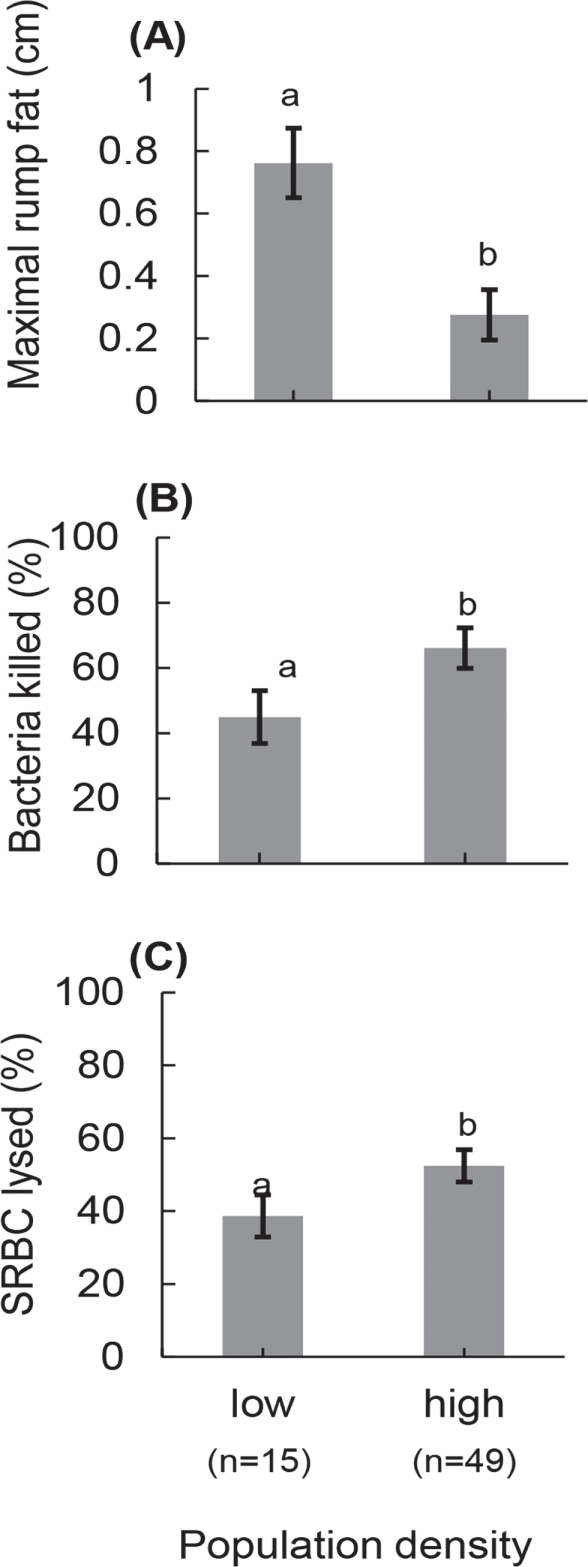
Constitutive immune responses and nutritional condition differed in North American elk maintained at different population densities. Estimated mean (± s.e.) (A) nutritional condition as indicated by maximal subcutaneous fat, (B) bacteria killing ability, and (C) hemolytic-complement activity by density for North American elk on the Starkey Experimental Forest and Range 2012–2013. Elk from the low-density population had higher bacteria killing ability, higher hemolytic-complement activity, and lower nutritional condition than elk from the high-density population. Population densities were relative to ecological carrying capacity. Different letters indicate significant differences (P < 0.05) of pairwise comparisons following analysis of variance.

**Fig 2:**
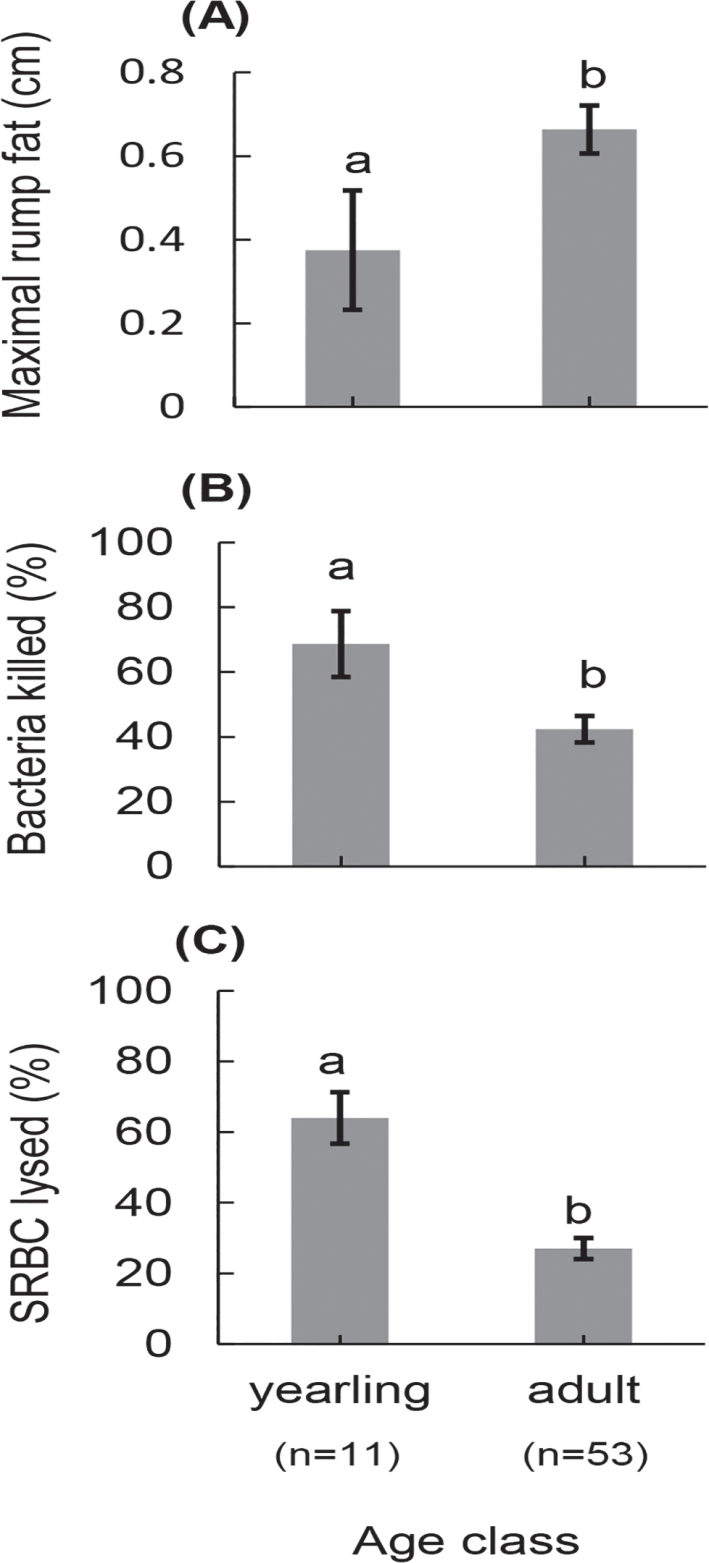
Constitutive immune responses and nutritional condition differed among adult and yearling North American elk. Estimated mean (± s.e.) (A) nutritional condition as indicated by maximal subcutaneous fat, (B) bacteria killing ability, and (C) hemolytic-complement activity for adult and yearling North American elk on the Starkey Experimental Forest and Range 2012–2013. Yearling elk had higher bacteria killing ability, higher hemolytic-complement activity and lower nutritional condition than adult elk. Different letters indicate significant differences (P < 0.05) of pairwise comparisons following analysis of variance.

**Fig 3:**
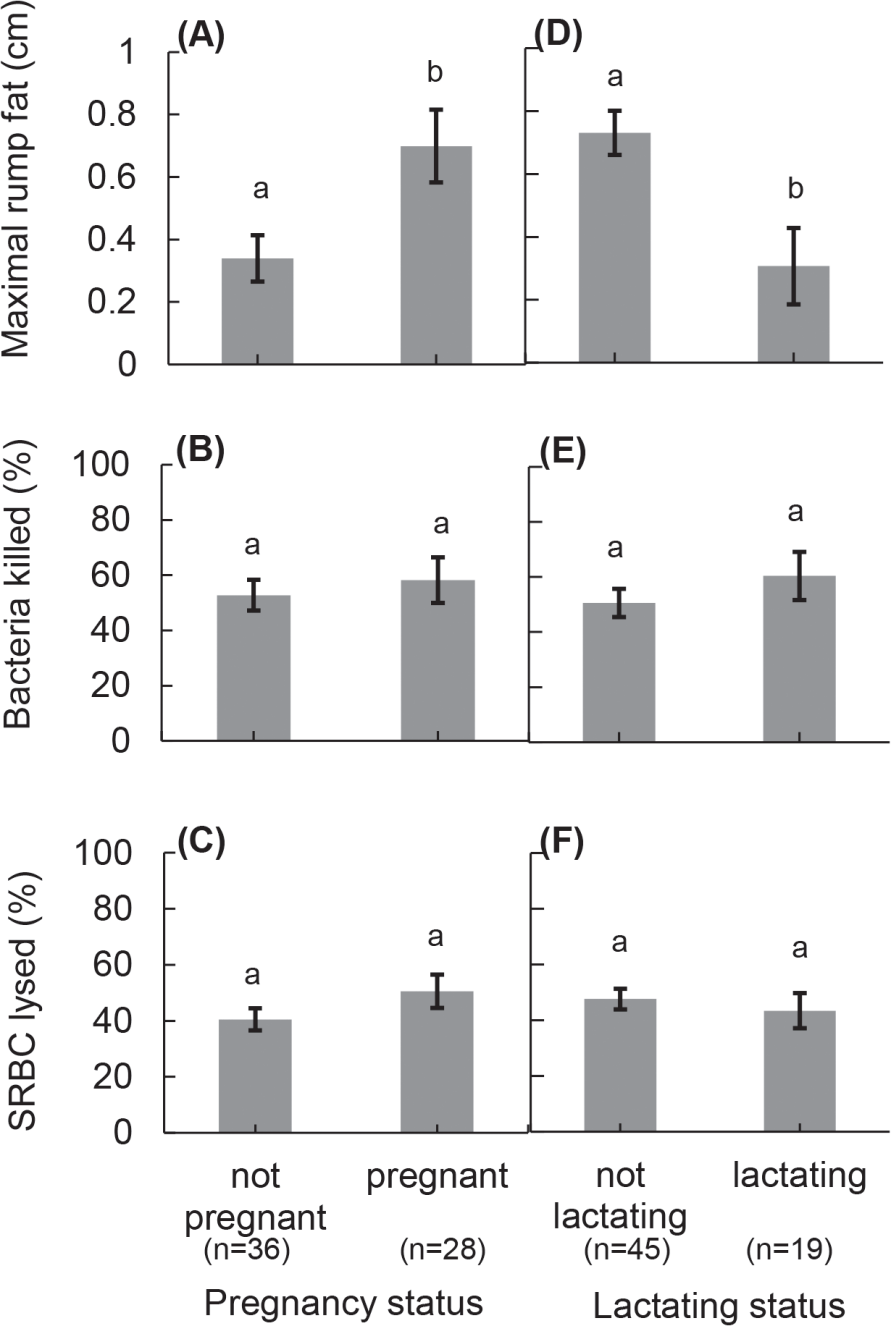
Constitutive immune responses and nutritional condition in pregnant and lactating North American elk. Estimated means (± s.e.) of (A) nutritional condition as indicated by maximal subcutaneous fat, (B) bacteria killing ability, and (C) hermolytic-complement activity by pregnancy status and (D) nutritional condition, (E) bacteria killing ability, and (F) hemolytic complement activity by lactation status for North American elk on the Starkey Experimental Forest and Range, 2012–2013. Pregnant elk were in higher nutritional condition than non-pregnant elk, but they did not differ in bacteria killing ability or higher hemolytic-complement activity. Lactating elk were in lower-nutritional condition than non-lactating elk, but did no differ in bacteria killing ability or hemolytic complement activity. Different letters indicate significant differences (P < 0.05) of pairwise comparisons following analysis of variance.

We included 64 female elk in our statistical analyses. Details of GLM model estimates for each response variable are located in S2 Table. Bacteria killing ability was significantly lower in the low-density population (n = 15) than the high-density population (n = 49, Wald *χ* ^*2*^_1_ = 6.23 *P* = 0.013; Fig 1B), and there was a pattern for higher bacteria killing ability in yearling elk (n = 11), when compared with adults (n = 53, Wald *χ* ^*2*^_1_ = 6.58, *P* = 0.010; Fig 2B). Bacteria killing ability did not differ according to pregnancy status (Wald *χ* ^*2*^_1_ = 0.46, *P* = 0.497; Fig 3B), lactation status (Wald *χ* ^*2*^_1_ = 1.31, *P* = 0.253; Fig 3E), or nutritional condition (β ± s.e. = 11.6± 8.8, Wald *χ* ^*2*^_1_ = 1.75, *P* = 0.186).

Hemolytic-complement activity was significantly lower in the low- (n = 15) than high-density population (n = 49, Wald *χ* ^*2*^_1_ = 5.12, *P* = 0.024; Fig 1C). Yearling elk (n = 11) had higher hemolytic-complement activity than adults (n = 53, Wald *χ* ^*2*^_1_ = 25.14, *P*< 0.001; Fig 2C). Hemolytic-complement activity did not differ by pregnancy status (Wald *χ* ^*2*^_1_ = 3.02, *P* = 0.082; Fig 3C) or lactation status (Wald *χ* ^*2*^_1_ = 0.46, *P* = 0.497; Fig 3F). Nutritional condition did not affect hemolytic-complement activity (β ± s.e. = 8.6 ± 6.3, Wald *χ* ^*2*^_1_ = 1.86, *P* = 0.173).

Nutritional condition differed significantly between population densities (Wald *χ* ^*2*^_1_ = 21.77, *P*<0.001; Fig 1A); individuals from the low-density population (n = 15) had 176% more fat than those from the high-density population (n = 49). Adults (n = 53) had subcutaneous fat that was 77% thicker than that of yearlings (n = 11, Wald *χ* ^*2*^_1_ = 4.16, *P* = 0.042; Fig 2A). Pregnant individuals (n = 28) had 105.8% thicker subcutaneous fat than non-pregnant individuals (n = 36, Wald *χ* ^*2*^_1_ = 11.56, *P*<0.001; Fig 3A). Those individuals that were not lactating (n = 45) had 138.3% thicker subcutaneous fat than those that were lactating (n = 19, Wald *χ* ^*2*^_1_ = 14.88, *P* <0.001; Fig 3D).

## Discussion

We did not observe any direct relationship between nutritional condition constitutive immunity. Although nutritional condition is known to help mediate investment in immunity [2, 61, 62], investment decisions depend on the type of nutritional deficit, for example fat versus protein. From our data we can make inferences about how fat reserves affected investment in immune function, but other types of nutritional limitations are beyond the range of our data. For example, important limiting factor in investment in immunity [63–65], and protein deficiencies generally suppress immune function [66], and reduce resistance to infections [67]. Thus, some specific nutrient, rather than energy levels measured as fat reserves, may be more important for determining investment in immune function. Alternatively, we may have failed to find a direct link between nutritional condition and constitutive immunity because nutritional condition also was associated with other potential predictors of immune function, including lactation status, pregnancy status, and population density.

Contrary to our hypothesis, elk in the high-density population had higher constitutive immunity than elk in the low-density population, which were in better nutritional condition. We suggest two potential explanations for the patterns we observed. First, elk in the high-density population may have invested more in constitutive immune responses because they were in poor nutritional condition. Thus they invested in defenses that prevent the establishment of a parasite infection and to prevent the need to mount the more costly induced immune response. Alternatively, parasite burdens may have differed between the two populations leading to differential activation of the immune system. These explanations are not mutually exclusive, and may interact synergistically to increase differences in investment in constitutive immunity that we observed between the two population densities.

The first potential explanation for our results is that elk in the high-density population invested more in constitutive immune responses because they were in lower nutritional condition and at greater physiological risk from diseases and parasites, as opposed to having a higher parasite burdens. This explanation may appear to contradict the straightforward idea that nutritional limitations lead to reductions of immune function [11, 68]. Nevertheless, relative costs and benefits depend on the type of immune response. We measured responses that are part of the humoral immune system, a system that primarily uses globular proteins to recognize, bind, and remove pathogens [38, 69]. Maintenance of constitutive humoral responses are generally less costly than activated cellular immune responses or humoral responses during an infection [9, 70, 71]. Thus, maintenance of constitutive humoral responses that quickly clear and prevent the establishment of a parasite might be the least costly immune strategy. It follows that individuals in poor nutritional condition may benefit from higher investment in the lower-cost constitutive immunity to prevent and clear infections quickly, rather than having to mount a high-cost immune response once an infection becomes established. Indeed, wild-caught mallards (*Anas platyrhynchos* Linnaeus) in poor nutritional condition had lower viral loads when experimentally inoculated with influenza A viruses than did wild-caught individuals in greater nutritional condition or captive mallards [72].

The second potential explanation for our results is that elk in different population densities were subject to different parasite burdens. Models of parasite transmission typically assume that transmission increases with population density, thus the likelihood of acquiring a parasite also increases with population density when parasites are transmitted directly among individuals (reviewed by [25, 26], but see exceptions therein). This assumption has general support in the literature [73, 74], and indeed diseases have been suggested as a mechanism through which density-dependent processes affect populations [75]. In addition, most research suggests that susceptibility to parasites increases with declining nutritional condition ([76, 77], but see Arsnoe et al. [69] who found the opposite pattern in mallards), resulting in a synergistic interaction between nutritional condition and transmission of diseases [78]. As a result, elk from the high-density population may have higher parasite loads than those from the low-density population, and thus might have higher immune responses because constitutive immune responses increase during an immunological challenge [79]. In this instance, our assay would not have just measured baseline levels of measured immune components, but also would have measured elevated levels of those immune responses caused by an induced immune response.

At present we cannot definitively distinguish between these two hypothesized explanations. Nevertheless, we suspect that that nutritional condition, rather than parasite load, is driving our results. Elk on Starkey have been monitored for brucellosis and Leptospira periodically. No sample has ever come back with positive titers for brucellosis and there are no obvious disease epidemics in this population, although Leptospira is endemic in the population (BLD personal observation). Furthermore, elk from both study areas were housed together on the winter feedground and foraged on hay together during winter (nose to nose contact is common), thus differences in parasite burden alone is unlikely because parasites could be easily transmitted between individuals from the high or low density populations on the winter feedground. Moreover, mortalities of individuals on the winter feedground are generally the result of poor nutritional condition prior to entering the feedground, rather than disease [17].

Yearling elk, in general, had lower subcutaneous fat than adults (Fig 1C). Young individuals often have smaller fat reserves in part because of their smaller body size [80]. In addition, differences in nutritional condition between yearlings and adults, also resulted from yearlings continuing to allocate resources to growth rather than storage of energy [43]. Yearling elk had both higher hemolytic-complement activity and higher bacteria killing ability than did adults, despite having lower energy reserves (Fig 2). Our results were consistent with a study of Iberian red deer (*Cervus elaphus hispanicus*), which reported that young born to food restricted females weighed less and had higher constitutive levels of antibodies, which indicated a negative relationship between nutritional condition and investment in constitutive immunity [81]. Our results with respect to age were also consistent with the explanation that nutritional condition dictated investment in constitutive immunity. Both our results concerning density and age group, the groups with the lowest fat reserves had the highest immune responses supporting the idea elk with lower fat reserves invest in frontline, constitutive immune responses that help prevent the establishment of parasites.

Results from the hemolytic-complement activity assay indicated that pregnancy did not alter investment in the complement pathway. Thus, pregnancy did not appear to draw resources away from investment in constitutive, complement activity. Similarly, Siberian hamsters (*Phodopus sungorus* Pallas) did not differ in bacteria killing ability based on pregnancy status [82], although fast-paced species tend to invest in reproduction over survival and often exhibit a trade-off between pregnancy and immune function [61, 62, 83–85]. Nevertheless, we measured immune function during the first trimester of pregnancy, which is less energetically demanding that later stages [80]. Indeed, other studies that examined the relationship between immune function and pregnancy measured immune function in later stages of reproduction when energetic investment in reproduction was greater. In Soay sheep (*Ovia aries*), individuals with higher indicators of high rates of division and antibody production by B and serum cells had a lower probability of successfully reproducing in females [86]. Hormones associated with pregnancy and the energy requirements of pregnancy are mechanistic explanations for trade-offs that have been found between investment in immune function and reproduction [3, 11, 87]. Those interactions, however, change over the course of pregnancy, and thus the likelihood of a tradeoff changes, and probably increases as pregnancy progresses. Those interactions create a complicated and dynamic relationship between immune function and reproduction [88], and more research into the mechanistic underpinnings of these results is required to illuminate how investment in constitutive immunity changes during pregnancy in free-roaming, slow-paced animals.

Elk in our study were captured during early winter, thus lactation status was a conservative index of successful recruitment of offspring [17, 42]. Our assessment of recruitment is a conservative index because some individuals that recruited an offspring may have stopped lactating prior to our sampling, whereas continuing to lactate after mortality of an offspring is unlikely [17, 42]. Lactating individuals had fewer fat reserves probably because of investment in reproduction, but did not alter levels of constitutive immunity (Fig 3F). Thus, our results also indicated the absence of a trade-off between investment in complement and reproductive success at this stage of the life cycle. At the time that we measured immune function in our study animals in early winter, the energetically expensive part of lactation when a neonate is provisioned solely by their mother’s milk had already occurred. Females in early winter likely were using lactation as a means of maintaining social bonds with their offspring [89], and lactation was primarily an indicator of survival of offspring and of recruitment. Thus, our experiment was really testing for lingering effects of the costs or reproduction on constitutive immunity, whereas most published results have focused on direct trade-offs between provisioning a neonate and immunity [11]. Although, our conclusion may appear to contradict most previous work indicating that provisioning offspring leads to decreased investment in immune function, measurement of immune function immediately after parturition likely is a better indicator of tradeoffs between immune function and reproduction [90–92].

## Conclusion

Comparisons of elk in high- and low-density populations indicated that as population density of elk increased, nutritional condition declined and individuals increased investment in constitutive immune function. Differences between individuals from different densities could have resulted because individuals in poor nutritional condition invested more in constitutive immune defenses, or because individuals from high-population densities had higher parasite loads causing induced immune responses. When considering differences in immune function and nutritional condition between individuals from different population densities and different age classes, our results provide stronger support for the hypothesis that animals in poorer nutritional condition invest more in constitutive immune defenses than animals in better nutritional condition. This strategy might allow elk in poorer condition to prevent the establishment of an infection and to avoid having to mount expensive adaptive immune responses. This intriguing hypothesis should be investigated further within the larger framework of the costs and benefits of immune responses.

Our results also speak to the effects of density dependence on individual-level traits. Density-dependent mechanisms are mediated through intraspecific competition, via per capita availability of food and the subsequent influence of nutrition on reproduction and recruitment of young into the reproductive segment of the population [17–20]. As populations approach carrying capacity and resources become increasingly limited, some individuals do not gain adequate fat levels for both survival and reproduction; as a result, limited fat stores are allocated to survival over reproduction in slow-paced species, including elk [22, 42, 93–96]. Elk from the high-density population invested more in immune function than those from the low-density population, supporting our hypothesis that immune function is a trait that is mediated by population density. Our results support the premise that density-dependent regulation of immune function is important for understanding population demographics of species that show strong density dependence in their life histories.

## Supporting Information

S1 Table Data used in analyses. (DOCX)

S2 Table Analysis of maximum likelihood parameter estimates for generalized linear models. Parameter estimates for generalized linear models to test for differences in bacteria killing ability, hemolytic-complement activity, and nutritional condition among elk at different population densities, from different age classes, with different lactation status, and with different pregnancy status. Nutritional condition was used as a covariate in models for bacteria killing ability and hemolytic complement activity.
(DOCX)

S1 Text A comparison of effects of storage methods of elk blood on immune assays. Methods and results for the experiment conducted to determine if freezing samples has a negative effect on bacteria killing ability and hemolytic-complement activity.
(DOCX)
