# Supplementary material for: Investment in Constitutive Immune Function by North American Elk Experimentally Maintained at Two Different Population Densities
Source: PLoS One. 2015 May 20;10(5):e0125586. doi: 10.1371/journal.pone.0125586 (PMC4439091; doi:10.1371/journal.pone.0125586)
Supplement: S1 Table — (DOCX) [file pone.0125586.s001.docx]

Supporting information for “Investment in Constitutive Immune Function by North American Elk Experimentally Maintained at Two Different Population Densities” by Downs, Stewart, and Dick

**S1 Table1. Data used in analyses.**

| % SRBC lysed | Age class^a^ | Lactational status | % e coli killed | Rump fat Max^b^ (cm) | Population density | Mass (kg) | Pregnancy status^c^ |
| --- | --- | --- | --- | --- | --- | --- | --- |
| 84.06 | YR | not lactating | 62.15 | 0.3 | High | 129.3 | NP |
| 39.53 | YR | not lactating | 64.83 | 0.29 | High | 147.9 | NP |
| 73.97 | YR | not lactating | 59.90 | 0.19 | High | 125.6 | NP |
| 39.39 | YR | not lactating | 78.19 | 0.18 | High | 144.7 | NP |
| 58.91 | YR | not lactating | 59.01 | 0.15 | High | 138.8 | NP |
| 82.19 | YR | not lactating | 73.01 | 0.23 | High | 133.4 | NP |
| 87.94 | YR | not lactating | 79.88 | 0.27 | High | 135.2 | NP |
| 86.82 | YR | not lactating | 85.65 | 0.14 | High | 126.1 | NP |
| 51.66 | YR | not lactating | 54.57 | 0.25 | High | 122.0 | NP |
| 39.13 | AD | not lactating | 75.20 | 0.56 | High | 180.5 | P |
| 11.64 | AD | not lactating | 15.64 | 0.2 | High | 181.4 | NP |
| 71.92 | AD | not lactating | 75.54 | 0.58 | High | 193.2 | P |
| 57.36 | AD | not lactating | 54.32 | 0.35 | High | 175.1 | NP |
| 82.17 | AD | not lactating | 63.88 | 1.43 | High | 205.5 | P |
| 31.78 | AD | lactating | 1.91 | 0.23 | High | 191.9 | NP |
| 23.84 | AD | not lactating | -3.15 | 0.43 | High | 202.3 | NP |
| 22.52 | AD | not lactating | 79.65 | 0.25 | High | 192.8 | NP |
| 11.26 | AD | lactating | 18.77 | 0.16 | High | 205.5 | NP |
| 13.95 | AD | not lactating | 40.25 | 0.18 | High | 203.2 | NP |
| 23.18 | AD | lactating | 78.64 | 0.23 | High | 197.3 | NP |
| 19.57 | AD | not lactating | 22.86 | 1.9 | High | 225.0 | P |
| 39.07 | AD | lactating | 81.08 | 0.47 | High | 212.3 | P |
| 50.68 | AD | lactating | 73.77 | 0.14 | High | 191.4 | P |
| 80.82 | AD | not lactating | 52.59 | 0.52 | High | 240.4 | P |
| 56.59 | AD | not lactating | 80.38 | 0.78 | High | 242.2 | P |
| 14.89 | AD | lactating | 61.47 | 0.2 | High | 195.0 | NP |
| 45.70 | AD | not lactating | 3.56 | 0.2 | High | 229.5 | P |
| 31.78 | AD | lactating | 88.04 | 0.2 | High | 225.4 | P |
| 21.71 | AD | not lactating | 11.00 | 0.32 | High | 211.4 | NP |
| 23.84 | AD | lactating | 54.43 | 0.18 | High | 198.7 | NP |
| 57.25 | AD | not lactating | 65.19 | 1.15 | High | 221.4 | P |
| 35.76 | AD | not lactating | 83.72 | 0.78 | High | 246.8 | P |
| 13.48 | AD | lactating | 29.38 | 0.15 | High | 200.9 | NP |
| 21.74 | AD | not lactating | 11.60 | 0.88 | High | 241.3 | P |
| 68.22 | AD | not lactating | 58.52 | 1.13 | High | 217.7 | P |
| 33.33 | AD | lactating | 40.91 | 0.4 | High | 201.4 | NP |
| 20.16 | AD | not lactating | 49.14 | 0.28 | High | 230.4 | P |
| 38.41 | AD | lactating | 75.28 | 0.15 | High | 181.9 | P |
| 45.74 | AD | not lactating | 83.49 | 1.33 | High | 226.8 | P |
| 13.48 | AD | lactating | 70.39 | 0.21 | High | 187.8 | NP |
| 78.81 | AD | not lactating | 39.01 | 0.35 | High | 200.9 | NP |
| 13.01 | AD | lactating | 28.63 | 0.24 | High | 216.4 | NP |
| 20.93 | AD | not lactating | 25.36 | 1.08 | High | 226.8 | P |
| 10.85 | AD | not lactating | -5.93 | 0.33 | High | 258.5 | P |
| 9.30 | AD | not lactating | 25.68 | 0.48 | High | 244.9 | P |
| 32.45 | AD | not lactating | 76.81 | 0.18 | High | 227.7 | P |
| 27.13 | AD | lactating | 74.64 | 0.15 | High | 195.0 | NP |
| 24.50 | AD | not lactating | 87.18 | 0.28 | High | 209.1 | NP |
| 13.18 | AD | not lactating | 75.12 | 0.14 | High | 196.0 | NP |
| 47.16 | YR | not lactating | 37.79 | 0.18 | Low | 133.8 | NP |
| 40.64 | YR | not lactating | 52.28 | 0.6 | Low | 182.3 | NP |
| 42.05 | AD | not lactating | 56.39 | 0.5 | Low | 167.8 | NP |
| 13.97 | AD | lactating | 6.95 | 0.2 | Low | 186.9 | NP |
| 9.63 | AD | not lactating | 29.22 | 1.58 | Low | 224.5 | P |
| 12.29 | AD | lactating | 4.99 | 0.45 | Low | 214.1 | NP |
| 63.64 | AD | not lactating | 61.19 | 1.98 | Low | 254.0 | P |
| 20.45 | AD | lactating | 46.00 | 0.45 | Low | 205.0 | NP |
| 28.34 | AD | not lactating | 58.33 | 1.23 | Low | 230.4 | P |
| 9.66 | AD | not lactating | 71.80 | 1.82 | Low | 238.6 | NP |
| 26.20 | AD | not lactating | 0.91 | 0.77 | Low | 205.5 | P |
| 28.34 | AD | not lactating | 13.70 | 1.43 | Low | 229.5 | P |
| 15.51 | AD | lactating | 35.05 | 0.23 | Low | 181.4 | NP |
| 57.95 | AD | not lactating | 60.96 | 2.15 | Low | 239.5 | P |
| 14.49 | AD | lactating | 32.18 | 0.33 | Low | 207.7 | P |

^a^ Age class is either yearling (YR) or adult (AD).

^b^ Maximal fat depth at the rump.

^c^ Pregnancy status is either pregnant (P) or not pregnant (NP).
