# Supplementary material for: Investment in Constitutive Immune Function by North American Elk Experimentally Maintained at Two Different Population Densities
Source: PLoS One. 2015 May 20;10(5):e0125586. doi: 10.1371/journal.pone.0125586 (PMC4439091; doi:10.1371/journal.pone.0125586)
Supplement: S2 Table — Parameter estimates for generalized linear models to test for differences in bacteria killing ability, hemolytic-complement activity, and nutritional condition among elk at different population densities, from different age classes, with different lactation status, and with different pregnancy status. Nutritional condition was used as a covariate in models for bacteria killing ability and hemolytic complement activity. (DOCX) [file pone.0125586.s002.docx]

Supporting information for “Investment in Constitutive Immune Function by North American Elk Experimentally Maintained at Two Different Population Densities” by Downs, Stewart, and Dick

**S2 Table. Analysis of maximum likelihood parameter estimates for generalized linear models.** Parameter estimates for generalized linear models to test for differences in bacteria killing ability, hemolytic-complement activity, and nutritional condition among elk at different population densities, from different age classes, with different lactation status, and with different pregnancy status. Nutritional condition was used as a covariate in models for bacteria killing ability and hemolytic complement activity. Sample size (n) indicated the number of elk in each category of each parameter.

| **Response variable** | **Parameter** |  | **n** | **DF^a^** | **Estimate** | **Standard Error** | **Wald 95% Confidence Limits** | | **Wald  Chi-Square** | **P** |
| --- | --- | --- | --- | --- | --- | --- | --- | --- | --- | --- |
| **Bacteria killing ability** | Intercept |  |  | 1 | 49.54 | 14.92 | 20.30 | 78.78 | 11.03 | <0.001 |
|  | Population density | High | 49 | 1 | 21.13 | 8.46 | 4.54 | 37.72 | 6.23 | 0.013 |
|  |  | Low | 15 | 0 | 0 | 0 | 0 | 0 | . | . |
|  | Age class | Adult | 53 | 1 | -26.3 | 10.25 | -46.40 | -6.21 | 6.58 | 0.010 |
|  |  | Yearling | 11 | 0 | 0 | 0 | 0 | 0 | . | . |
|  | Lactation status | lactating | 19 | 1 | 9.80 | 8.57 | -6.99 | 26.60 | 1.31 | 0.253 |
|  |  | Not lactating | 45 | 0 | 0 | 0 | 0 | 0 | . | . |
|  | Pregnancy status | Not Pregnant | 36 | 1 | -5.47 | 8.05 | -21.2 | 10.31 | 0.46 | 0.497 |
|  |  | Pregnant | 28 | 0 | 0 | 0 | 0 | 0 | . | . |
|  | Nutritional condition^b^ |  | 64 | 1 | 11.60 | 8.77 | -5.59 | 28.80 | 1.75 | 0.186 |
|  | Scale |  |  | 1 | 24.72 | 2.18 | 20.79 | 29.39 |  |  |
|  |  |  |  |  |  |  |  |  |  |  |
| **Hemolytic-complement activity** | Intercept |  |  | 1 | 59.55 | 10.73 | 38.51 | 80.59 | 30.77 | <0.001 |
|  | Population density | High | 49 | 1 | 13.79 | 6.09 | 1.86 | 25.73 | 5.13 | 0.0235 |
|  |  | Low | 15 | 0 | 0 | 0 | 0 | 0 | . | . |
|  | Age class | Adult | 53 | 1 | -37.00 | 7.38 | -51.46 | -22.54 | 25.14 | <0.001 |
|  |  | Yearling | 11 | 0 | 0 | 0 | 0 | 0 | . | . |
|  | Lactation status | lactating | 19 | 1 | -4.19 | 6.17 | -16.28 | 7.90 | 0.46 | 0.497 |
|  |  | Not lactating | 45 | 0 | 0 | 0 | 0 | 0 | . | . |
|  | Pregnancy status | Not Pregnant | 36 | 1 | -10.08 | 5.80 | -21.44 | 1.29 | 3.02 | 0.0822 |
|  |  | Pregnant | 28 | 0 | 0 | 0 | 0 | 0 | . | . |
|  | Nutritional condition^b^ |  | 64 | 1 | 8.60 | 6.31 | -3.77 | 20.97 | 1.86 | 0.173 |
|  | Scale |  |  | 1 | 17.79 | 1.57 | 14.96 | 21.15 |  |  |
|  |  |  |  |  |  |  |  |  |  |  |
| **Nutritional condition^b^** | Intercept |  |  | 1 | 1.01 | 0.17 | 0.67 | 1.34 | 34.83 | <0.001 |
|  | Population density | High | 49 | 1 | -0.49 | 0.10 | -0.69 | -0.28 | 21.77 | <0.001 |
|  |  | Low | 15 | 0 | 0 | 0 | 0 | 0 | . | . |
|  | Age class | Adult | 53 | 1 | 0.29 | 0.14 | 0.01 | 0.57 | 4.16 | 0.042 |
|  |  | Yearling | 11 | 0 | 0 | 0 | 0 | 0 | . | . |
|  | Lactation status | lactating | 19 | 1 | -0.42 | 0.11 | -0.64 | -0.21 | 14.88 | <0.001 |
|  |  | Not lactating | 45 | 0 | 0 | 0 | 0 | 0 | . | . |
|  | Pregnancy status | Not Pregnant | 36 | 1 | -0.36 | 0.11 | -0.57 | -0.15 | 11.56 | <0.001 |
|  |  | Pregnant | 28 | 0 | 0 | 0 | 0 | 0 | . | . |
|  | Scale |  |  | 1 | 0.35 | 0.03 | 0.30 | 0.42 |  |  |

^a^  DF = degrees of freedom.

^b^ Nutritional condition was quantified by measuring maximal depth of subcutaneous fat on the rump.
