# Supplementary material for: Investment in Constitutive Immune Function by North American Elk Experimentally Maintained at Two Different Population Densities
Source: PLoS One. 2015 May 20;10(5):e0125586. doi: 10.1371/journal.pone.0125586 (PMC4439091; doi:10.1371/journal.pone.0125586)
Supplement: S1 Text — Methods and results for the experiment conducted to determine if freezing samples has a negative effect on bacteria killing ability and hemolytic-complement activity. (DOCX) [file pone.0125586.s003.docx]

Supporting information for “Investment in Constitutive Immune Function by North American Elk Experimentally Maintained at Two Different Population Densities” by Downs, Stewart, and Dick

**S1 Text. A comparison of effects of storage methods of elk blood on immune assays.** Methods and results for the experiment conducted to determine if freezing samples has a negative effect on bacteria killing ability and hemolytic-complement activity.

We tested a separate set of 10 samples from elk to determine if freezing the samples affected results of immunocompetence assays as reported in previous work quantifying bacteria killing ability in house sparrows (*Passer domesticus* Linnaeus) [1]. We collected blood in the manner described in the main article (“*Study Area and Study Design*”). Each sample was partitioned into two aliquots; one aliquot was frozen and the other refrigerated. After three days, the time required to transport the samples from the field to the laboratory, we conducted immunocompetence assays for both refrigerated and frozen samples. Assays are described in detail in the main text of the article (“*Immunocompetence Assays*”)

To determine the effect of freezing serum on the two immune assays used in this study, we compared the percentage of bacteria killed in the bacteria killing assay and the percentage of sheep red blood cells lyses in the hemolytic-complement activity assay using general linear mixed models. The fixed effect was sample storage (refrigerator or freezer). We did not include any other parameters as fixed effects, because we were only interested in whether freezing decreased the viability of our serum samples. We accounted for variation among individuals by including the individual as a random effect in the analysis. We developed general linear mixed models using procedure lme in program R version 3.0.0 [2].

Samples stored in the freezer had higher bacteria killing ability (*F*_1,9_ = 28.07, *P* = 0.034) and higher hemolytic-complement activity (*F*_1,9_ = 7.78, *P* < 0.021) than samples stored in the refrigerator, indicating that freezing does not degrade elk serum samples, and that the samples degraded significantly in the refrigerator in the three days required to transport the samples from the field site to the laboratory.

1. Liebl AL, Martin LB (2009) Simple quantification of blood and plasma antimicrobial capacity using spectrophotometry. Functional Ecology 23: 1091-1096.
2. R Development Core Team (2013) R: A language and environment for statistical computing. R Foundation for Statistical Computing, Vienna, Austria.
